# Collagen Fiber Array of Peritumoral Stroma Influences Epithelial-to-Mesenchymal Transition and Invasive Potential of Mammary Cancer Cells

**DOI:** 10.3390/jcm8020213

**Published:** 2019-02-07

**Authors:** Marco Franchi, Valentina Masola, Gloria Bellin, Maurizio Onisto, Konstantinos- Athanasios Karamanos, Zoi Piperigkou

**Affiliations:** 1 Department for Life Quality Studies, University of Bologna, Rimini 47100, Italy; 2 Renal Unit, Department of Medicine, University Hospital of Verona, Verona 37100, Italy; valentina.masola@unipd.it (V.M); gloria.bellin@gmail.com (G.B); 3 Department of Biomedical Sciences, University of Padova, Padua 35100, Italy; maurizio.onisto@unipd.it; 4 Department of Pharmacy and Industrial Pharmacy, University of Bologna, Bologna 40100, Italy; karamanos.akis@gmail.com; 5 Biochemistry, Biochemical Analysis & Matrix Pathobiology Research Group, Laboratory of Biochemistry, Department of Chemistry, University of Patras, Patras 26110, Greece; zoipip@upatras.gr

**Keywords:** breast cancer, extracellular matrix, collagen type I, scanning electron microscope, epithelial-to-mesenchymal transition

## Abstract

Interactions of cancer cells with matrix macromolecules of the surrounding tumor stroma are critical to mediate invasion and metastasis. In this study, we reproduced the collagen mechanical barriers in vitro (i.e., basement membrane, lamina propria under basement membrane, and deeper bundled collagen fibers with different array). These were used in 3D cell cultures to define their effects on morphology and behavior of breast cancer cells with different metastatic potential (MCF-7 and MDA-MB-231) using scanning electron microscope (SEM). We demonstrated that breast cancer cells cultured in 2D and 3D cultures on different collagen substrates show different morphologies: i) a globular/spherical shape, ii) a flattened polygonal shape, and iii) elongated/fusiform and spindle-like shapes. The distribution of different cell shapes changed with the distinct collagen fiber/fibril physical array and size. Dense collagen fibers, parallel to the culture plane, do not allow the invasion of MCF-7 and MDA-MB-231 cells, which, however, show increases of microvilli and microvesicles, respectively. These novel data highlight the regulatory role of different fibrillar collagen arrays in modifying breast cancer cell shape, inducing epithelial-to-mesenchymal transition, changing matrix composition and modulating the production of extracellular vesicles. Further investigation utilizing this in vitro model will help to demonstrate the biological roles of matrix macromolecules in cancer cell invasion in vivo.

## 1. Introduction

Cancer cell invasion and intravasation are the initial steps of metastasis during tumor progression [1,2,3]. Extracellular matrix (ECM), the main component of connective tissues, is a physical scaffold, which must adapt to deformations caused by internal and external mechanical stresses. In particular, the fibrillar protein component (collagen and elastin) of ECM transmits and resists tensional forces, whereas the hydrophilic and water-soluble main components of the ground substance (glycosaminoglycans and proteoglycans) resist compressive forces [4,5]. Under normal conditions, the different density of ECM functionally allows the diffusion of oxygen and nutrients and modulates the resistance that moving cells encounter crossing the connective tissue. Interactions between primary tumor cells and stroma microenvironment are related to both biomechanical and biological mechanisms. Besides the remodeling of the collagen fiber network of tumor stroma around the tumor mass, some ECM molecules, such as hyaluronan (HA), proteoglycans, and glycoproteins (e.g., fibrin, fibronectin and vitronectin), are upregulated and become able to biologically affect stroma cells [6]. Apart from HA, collagen VI upregulation modulates tumor metastasis in the stroma microenvironment of breast cancer [7]. On the other hand, collagen XV in basal membrane disappears prior to tumor metastasis in several organs, confirming its role in maintaining basement membrane integrity and preventing the first step of tumor cell invasion [8]. An important biological interaction between tumor and stroma is also represented by the ability of cancer cells to release extracellular vesicles, which are small plasma membrane-derived particles released into the extracellular space and differ in their size. Extracellular vesicles, including both the smaller exosomes (30–100 nm) and the larger microvesicles (100–1000 nm) represent one of the structural and functional components of normal ECM; they affect matrix organization, regulation of cells within it, and physical properties of soft connective tissues, bone, cartilage and dentin [9,10,11]. In cancer, they contain second messengers, matrix metalloproteinases (MMPs), syndecans, HA, allow communications between cancer cells and stroma cells, and play a role in favoring cancer cell invasion and metastasis [12,13,14,15].

In breast tumor stroma, the collagen fiber network has an important role as a mechanical barrier to limit tumor cell migration and invasion. When the curly and smooth collagen fibers surrounding normal epithelial ducts become denser, more aligned and stiffer around the mammary tumor, the risk of metastasis is higher [16,17,18,19,20]. ECM stiffening during tumor development is related to deposition of fibronectin, proteoglycans, collagens I, III, IV and increased matrix cross-linking [21,22].

In connective tissues, the collagen fiber network represents a physical barrier, but when cancer cell invasion in tumor stroma occurs, the cancer cells can easily cross ECM by two different movements: the ameboid movement and the mesenchymal movement, which includes single or collective migration [23]. Both the individual mesenchymal invasion of single spindle-shaped cancer cells (mesenchymal shape, similar to fibroblast shape) and the collective mesenchymal invasion with one or more leader cells, generating forward traction and pericellular proteolysis toward the tissue structure, require collagen degradation by MMPs [23]. In contrast, proteolysis and breakdown of the ECM collagen barrier is not necessary in single/ameboid migrating cells, which are able to adapt their shape in order to penetrate the tumor stroma through small tissue gaps between collagen fibers [6].

ECM remodeling and stiffening are mechanical and biological events, which increase the stroma resistance to assault of pH and proteolytic degradation [24,25], and include a protease-dependent collagen cross-linking mechanism, which is related to lysyl oxidase (LOX) secretion by hypoxic tumor cells, but also a non-enzymatic collagen cross-linking process, which is related to glycation and transglutamination or to an increased deposition of proteoglycans [26]. However, the LOX-mediated collagen cross-linking process activated by hypoxic tumor cells seems to favor stromal cells with respect to adhering and secreting more MMPs, further promoting the tumor invasion by degrading collagen [27,28]. Tumor invasion is related to the epithelial-to-mesenchymal transition (EMT) of tumor cells, which occurs when cancer cells gain a spindle-like or mesenchymal shape, lose their adhesive properties and increase their motility and invasive ability [29,30]. Mechanical tension of collagen fibers around the tumor mass initially limits tumor expansion and cancer cell invasion; however, due to increased pressure, both cancer and stromal cells respond by increasing the drawing force on collagen fibers. This traction on ECM deforms them through integrin binding and allows the penetration of MMPs among collagen fibers which are degraded [31,32]. An increasing mechanical force transmitted by collagen fibers may also generate a change in basal membrane viscosity, thus altering the basal membrane permeability to macromolecules and cells [33].

In general, the architecture of peritumoral collagen is severely altered, and collagen remodeling seems to be highly relevant to cancer progression [34]; thus, focus is given to the classification of different arrays of peritumoral collagen fibers as markers of mammary carcinoma progression; the Tumor-Associated-Collagen-Signatures (TACS) [35]. Increased deposition of collagen around the mammary tumor occurs in the first step (TACS-I), but when tumor increases in size a straightening and an alignment of collagen fibers running parallel to the tumor surface comes about (TACS-II). The remodeling of the stroma leads to the final stage, characterized by collagen fibers arranged perpendicularly to the tumor boundary (TACS-III). This stage seems strictly related to a higher risk of cancer cell invasion and metastasis, because tumor cells preferentially invade along straightened, stiff and radially aligned collagen fibers [36,37]. Interestingly, biomechanical studies suggest that matrix topography, rather than tissue stiffness, is the dominant feature by which an aligned matrix can enhance invasion through 3-dimensional (3D) collagen matrices by durotaxis [38].

Several reports suggest that cancer stroma microenvironment has an epigenetic influence on the behavior and differentiation of cancer cells. For instance, cancer-associated fibroblasts (CAFs), which are normal fibroblasts activated by primary tumor cells, control morphology, promote proliferation, metastasis and drug resistance of cancer cells [39,40,41]. The primary breast tumor shows evident structural changes like desmoplasia and progressive changes of the array of collagen fibers, which may influence the behavior of cancer cells through epigenetic mechanisms [24,42].

Our hypothesis is that the different collagen array of natural barriers and changes of collagen fiber orientation in peritumoral stroma could influence the shape, the cytoplasmic morphology and the biological behavior of primary cancer cells. Therefore, the aim of this study was to mimic the different natural collagen barriers (basal membrane lamina propria under basement membrane with random fibrillar collagen array and dermis bundled collagen fibers with different array), which breast cancer cells come across during the in vivo invasion of stroma microenvironment, in vitro. These reproduced barriers were used in 3D cultures to investigate how these different substrates affect the phenotype and invasive potential of different breast cancer cells (MCF-7 cells of low aggressiveness and MDA-MB-231 of high aggressiveness) using scanning electron microscope (SEM).

## 2. Materials and Methods

### 2.1. Cell Cultures

MDA-MB-231 and MCF-7 breast cancer cells (obtained from the American Type Culture Collection (ATCC)) were cultured in DMEM-High Glucose (EuroClone) (17.5 mM glucose) supplemented with 10% fetal bovine serum (FBS) (Biochrom AG), L-glutamine (2 mM), penicillin (100 U/mL), and streptomycin (100 μg/mL), at 37 °C in a humidified atmosphere with 5% CO_2_. MDA-MB-231 and MCF-7 breast cancer cells were seeded in flasks for 2D cultures and in different culture substrates (Millipore filter, Matrigel, collagen fibril network, collagen membrane) for 3D cultures. 2D cultures in polystyrene flasks and 3D cultures in a “Isopore Membrane Filter” with a pore size of 8.0 μm (Millipore, Milan, Italy) were used as control groups. In 3D cultures, the Millipore filter was coated with 50 μL of Matrigel solutions (0.18 μg/μL and 3.0 μg/μL) (BD Biosciences, Milan, Italy) so as to reproduce the natural basement membrane. This mimics the barrier, since Matrigel contains macromolecular components similar with the natural barrier (laminin, nidogen and heparan sulfate proteoglycans). Similarly, to mimic the sub-basal reticular fibrillar network of fibrillar collagen, a Millipore filter was coated with 50 μL of collagen type I solution (0.2 μg/μL). To reproduce the different array of collagen fibers in the peritumoral microenvironment reported during breast cancer development [43], a type I collagen membrane with fibers array parallel to the plane of the culture (Bioteck, Milan, Italy) and a type I collagen membrane with fibers arranged orthogonally to the plane of the culture (Condress, EURORESEARCH, Milan, Italy) were used.

### 2.2. Scanning Electron Microscopy

MDA-MB-231 and MCF-7 breast cancer cells seeded in flasks for 2D cultures and in different culture substrates (Millipore filter, Matrigel, collagen, collagen membrane) for 3D cultures were fixed in a Karnovsky’s solution for 20 min. Flasks and different culture substrates with adhered cells were rinsed three times with 0.1% cacodylate buffer, post-fixed in 1% OsO_4_ in cacodylate buffer for 20 min, dehydrated with increasing concentrations of ethanol, and finally dehydrated via with hexamethyldisilazane (Sigma-Aldrich, Inc.) for 15 min. The specimens were mounted on appropriate stubs, coated with a 5 nm palladium gold film (Emitech 550 sputter-coater) to be observed under a SEM (Philips 515, Eindhoven, The Netherlands) operating in secondary-electron mode.

### 2.3. Invasion Test and Statistical Analysis

The invasive behavior of MDA-MB-231 and MCF-7 cells was assessed by the Boyden-chamber assay, according to the method of Albini et al. [44], with slight modifications. The filters used in these assays were “Isopore Membrane Filters” (Millipore, Milan, Italy) with pore size of 8.0 μm. The filters were used as such or coated with 50 μL of Matrigel solution (0.18 μg/μL and 3.0 μg/μL) (BD Biosciences, Milan, Italy) or with 50 μL of type I collagen solution (0.2 μg/μL). It is mentioned that the density of collagen could not be increased in this experimental approach, since the thickness of the collagen membranes does not allow counting of the cells passing through the membranes. 10^5^ cells were resuspended in 800 μL of DMEM serum-free culture medium and were layered on top of the filters, in the upper compartment of the chamber, while in the lower compartment, DMEM medium supplemented with 20% FBS was used as chemoattractant. After 5-h incubation at 37 °C, cells that remained in the upper part of Matrigel and collagen type I filters were removed. Filters were washed in water, fixed in 100% ethanol for 5 min and stained with 1% toluidine blue/1% sodium tetraborate for 4 min. Following staining, filters were let to dry and photographed using Canon PowerShot G6 camera. Images were analyzed by ImageJ software (http://rsb.info.nih.gov/ij/).

### 2.4. RNA Isolation and Real-Time qPCR analysis

Breast cancer cells were seeded in Boiden chambers on 8.0 μm pore size Millipore filters and in filters coated with 50 μL of collagen type I solution (0.2 μg/μL). Total mRNA was extracted using the Trizol reagent (Invitrogen), following the manufacturer’s instructions. Quantity and quality of RNA were checked using the Nanodrop spectrophotometer (EuroClone). Total RNA was reverse-transcribed into cDNA using the reverse transcriptase SuperScript II (Invitrogen). Real-time qPCR was performed with the ABI-Prism 7500 using Power SYBR Green Master Mix 2 (Applied Biosystems) and specific primers for alpha-smooth muscle actin (α-SMA), E-cadherin, fibronectin, vimentin, MMP-2, MMP-9 and glyceraldehyde-3-phosphate dehydrogenase (GAPDH) (Table 1). The comparative Ct method (ΔΔCt) was used to quantify gene expression, and the relative quantification was calculated as 2^-ΔΔCt^. GAPDH gene amplification was used as a reference standard to normalize the target signal. Amplification specificity was controlled by melting curve analysis.

### 2.5. Statistical Analysis

Reported values are expressed as mean ± standard deviation (SD) of experiments performed in triplicate. Statistically significant differences were evaluated using the analysis of two-tailed Student’s *t*-test and were considered statistically significant at the level of at least *p* ≤ 0.05. Statistical analysis was performed using Rest2009 software.

## 3. Results

### 3.1. Breast Cancer Cell Morphology in 2D Cultures

**Figure 1:**
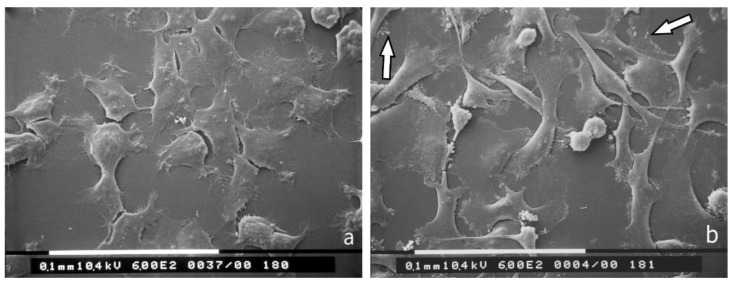
2D cultures in polystyrene flasks of breast cancer cells observed at SEM. (**a**) Most of the MCF-7 cells appear grouped with many cell-cell contacts and show a flattened, smoothly polygonal shape with very few cytoplasmic microvilli and rare cytoplasmic vesicles. Bar 100 μm; (**b**) MDA-MB-231 cells are presented as isolated cells with very few cell-cell contacts. These cells appear as globular/spherical cells, flattened elongated cells and spindle-like cells with a few cytoplasmic vesicles (arrows). Bar 100 μm.

SEM ultrastructural analysis of MCF-7 breast cancer cells cultured in polystyrene flasks demonstrated that most of the cells appeared grouped with many cell-cell contacts. Smooth cells in 2D cultures exhibited a flattened, polygonal shape with very few cytoplasmic microvilli and rare cytoplasmic vesicles (Figure 1a). On the other hand, MDA-MB-231 cells looked like isolated cells with few cell-cell contacts, characteristic of aggressive mesenchymal cancer cells. They included equally distributed globular/spherical cells, flattened-elongated cells and spindle-like cells showing filopodia, lamellipodia and cytoplasmic vesicles (Figure 1b).

### 3.2. 3D Cultures on Millipore Filter

**Figure 2:**
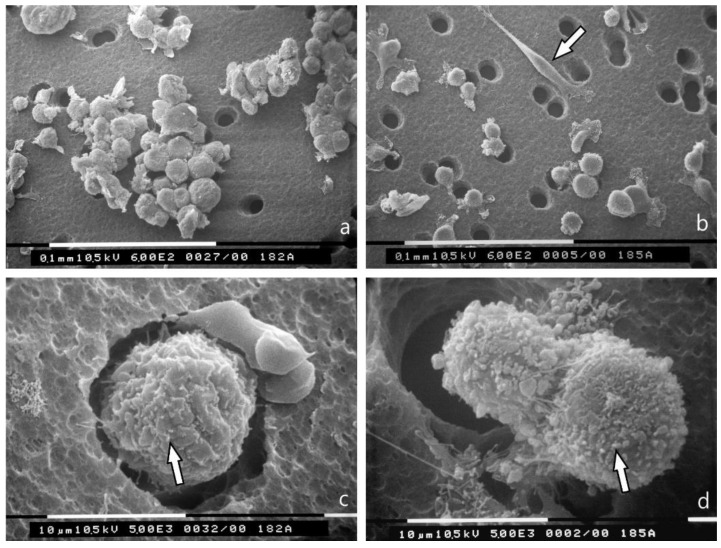
3D Millipore filter cultures observed at SEM. (**a**) MCF-7 cells assemble around the holes of the Millipore filter with many cell-cell contacts and show a globular/spherical morphology with a diameter of about 10 μm. Bar 100 μm; (**b**) Most of the MDA-MB-231 cells have a globular/spherical shape with a mean diameter of 10 μm and evident cytoplasmic microvesicles. An elongated and spindle-like cell (arrow) with very few microvilli and microvesicles is also visible. Bar 100 μm; (**c**) A globular/spherical cell is passing through a hole of Millipore filter and exhibits few microvilli (arrow) but no microvesicles. Bar 10 μm; (**d**) Two MDA-MB-231 globular cells with microvesicles (arrow) are crossing the holes of the Millipore filter. Bar 10 μm.

MCF-7 cells in 3D cultures on a Millipore filter as substrate completely changed their phenotype, as almost all of them showed a globular/spherical shape with a mean diameter of about 10 μm. Cells showed few microvilli, but no microvesicles, and were grouped in tight contacts while migrating into the holes of the Millipore filter (Figure 2a,c). Most of MDA-MB-231 cells cultured on Millipore filter displayed a globular/spherical shape with a mean diameter of 10 μm, and evident cytoplasmic microvesicles. Few elongated and spindle-like cells crossing the holes of Millipore filter, with filopodia and lamellipodia, few microvilli and microvesicles were also detectable (Figure 2b,d).

### 3.3. 3D Cultures on Millipore Filter Covered with Various Matrigel Concentrations

**Figure 3:**
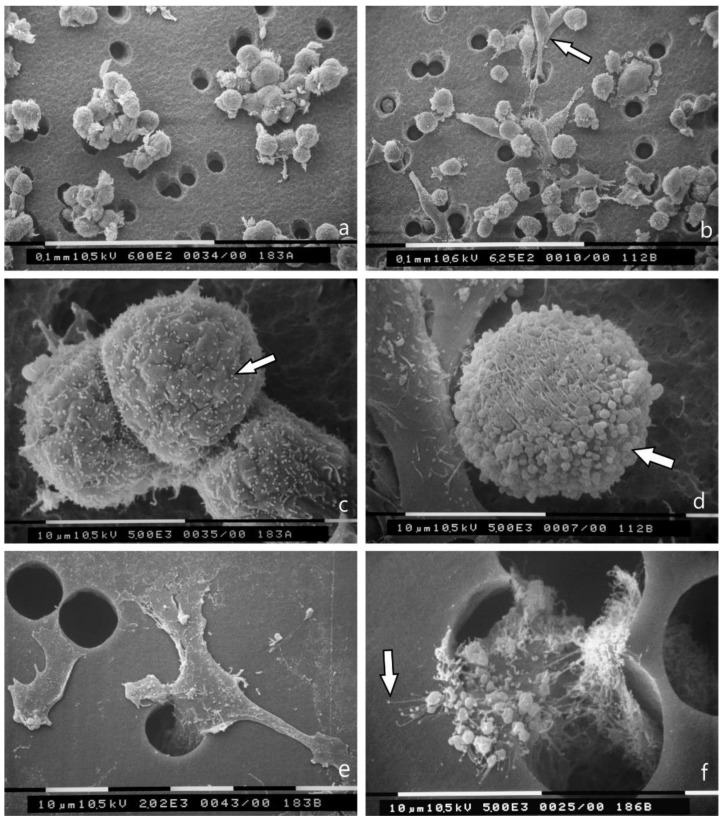
3D Matrigel (0.18 μg/mL) cell cultures as observed at SEM. (**a**) MCF-7 cells cultured on Matrigel are grouped and pass through the holes of Millipore filter. Bar 100 μm; (**b**) Most of the MDA-MB-231 cells show a globular/spherical shape and are rich in superficial microvesicles but elongated /spindle-like cells are also visible (arrow). Bar 100 μm; (**c**) MCF-7 cells show globular/spherical shape with cell-cell contacts and microvilli (arrow) on the cytoplasmic surface. Bar 100 μm; (**d**) A globular/spherical MDA-MB-231cell shows microvilli and many microvesicles (arrow). On the left, an elongated, flattened cell with few microvilli and microvesicles is detectable. Bar 10 μm; (**e**) In the lower side of the Millipore filter, MCF-7 cells passed through the holes do not show microvesicles but few microvilli. Bar 10 μm; (**f**) In the lower surface of the Millipore filter one MDA-MB-231 cell is passing through a filter hole and shows many microvesicles and microvilli generating exosomes (arrow). Bar 10 μm.

Breast cancer cell cultures on Millipore filter covered with Matrigel (0.18 μg/mL) and observed at SEM showed that MCF-7 cells are grouped while passing through the holes of Millipore filter. They exhibited a globular/spherical shape and microvilli on the cytoplasmic surface, which were visible also on cells have passed through the Millipore filter holes still exhibited microvilli (Figure 3a,c,e). On the other hand, MDA-MB-231 cells showed a mixed population of both globular/spherical and elongated/spindle-like cells with filopodia and lamellipodia. The predominating globular cells were richer in microvesicles and exosomes, also after crossing the Millipore holes (Figure 3b,d,f).

**Figure 4:**
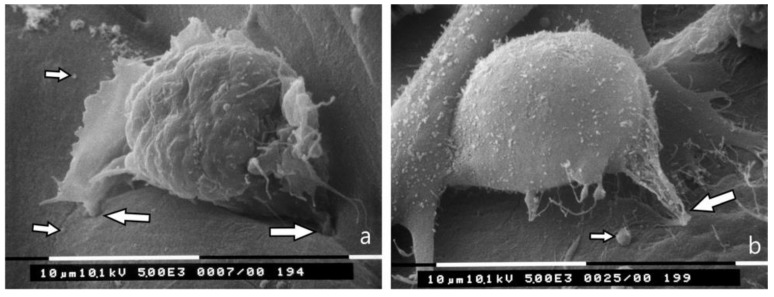
3D Matrigel (3.0 μg/mL) cell cultures analyzed by SEM. (**a**) MCF-7 cell lying on a thick layer of Matrigel shows very few microvilli and no microvesicles. Two invadopodia penetrating the Matrigel develop from the ventral side of the cell (large arrows). A few exosomes and microvesicles are shed on the Matrigel surface (small arrows). Bar 10 μm; (**b**) An MDA-MB-231 cell with few microvilli and microvesicles shows invadopodia (large arrow) penetrating into the thickness of Matrigel. Two adjacent elongated cells with few microvesicles are also visible. Few exosomes and microvesicles (small arrow) shed from MDA-MB-231 cells are present on the Matrigel surface next to the cells. Bar 10 μm.

When the thickness of the Matrigel coating on the Millipore filter was increased to 3 μg/mL, some of the MCF-7 and MDA-MB-231 cells with few microvilli and microvesicles showed evident invadopodia, penetrating the thickness of the Matrigel barrier. A few exosomes shed from MCF-7 cells and microvesicles shed from MDA-MB-231 cells were also observable on the Matrigel surface next to the cells (Figure 4a,b).

### 3.4. Breast Cancer Cell Cultures on Randomly Arranged Type I Collagen Fibril Network

**Figure 5:**
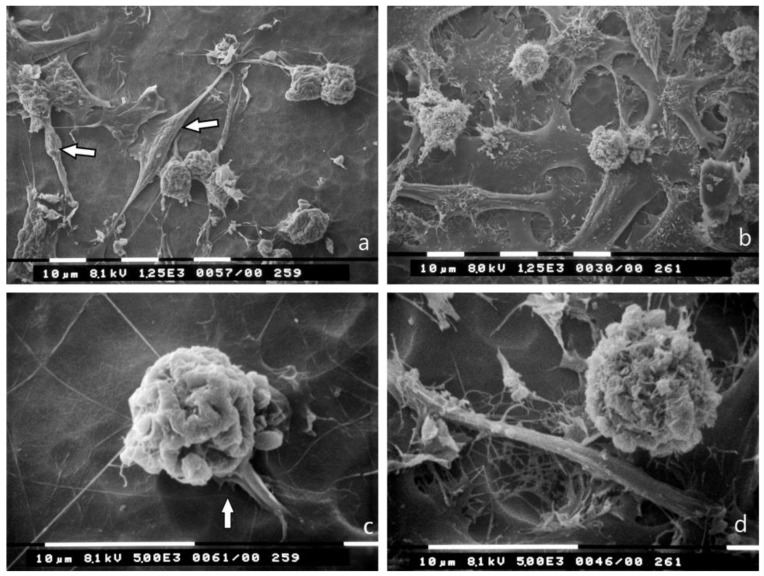
Breast cancer cells cultured on type I collagen fibrils (lamina propria under basement membrane) observed at SEM. The randomly arranged collagen fibrils completely occlude the holes of the Millipore filter. (**a**) Several MCF-7 cells showing both globular/spherical and flattened elongated shapes appear isolated or relatively grouped. A few spindle-like cells are also detectable (arrows). Bar 10 μm; (**b**) MDA-MB-231 breast cancer cells show both globular/spherical and flattened elongated shapes. Bar 10 μm; (**c**) A globular/spherical MCF-7 cell shows cytoplasmic convolutions, very few microvilli and no microvesicles. Invadosomes from the cell ventral surface are also visible (arrow). Bar 10 μm; (**d**) A globular/spherical MDA-MB-231 cell in proximity of a Millipore hole covered by sparse collagen fibrils shows microvesicles. Bar 10 μm.

Cultures of MCF-7 and MDA-MB-231 breast cancer cells on a Millipore filter were covered by randomly arranged collagen fibrils, representing lamina propria under basement membrane, almost completely occluding the holes of the Millipore filter showed increased cell proliferation. Similarly, grouped MCF-7 and MDA-MB-231 cells, included both globular/spherical and flattened elongated cells with filopodia and lamellipodia. Interestingly, spindle-like cells were also detectable in both groups (Figure 5a,b). MCF-7 cells showed cytoplasmic convolutions and very few microvilli; hence, invadosomes were also present (Figure 5c). Microvesicles or blebs were exclusively detectable in globular/spherical MDA-MB-231 cells (Figure 5d).

### 3.5. Bundled Type I Collagen Fibers Parallel to the Plane of the Culture (TACS II)

**Figure 6:**
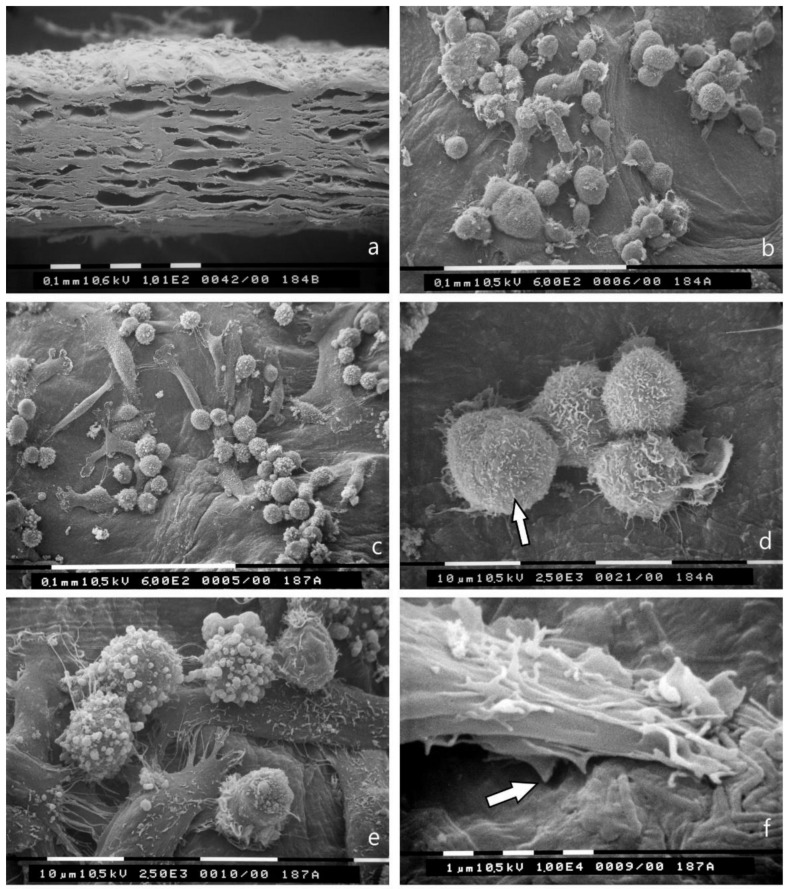

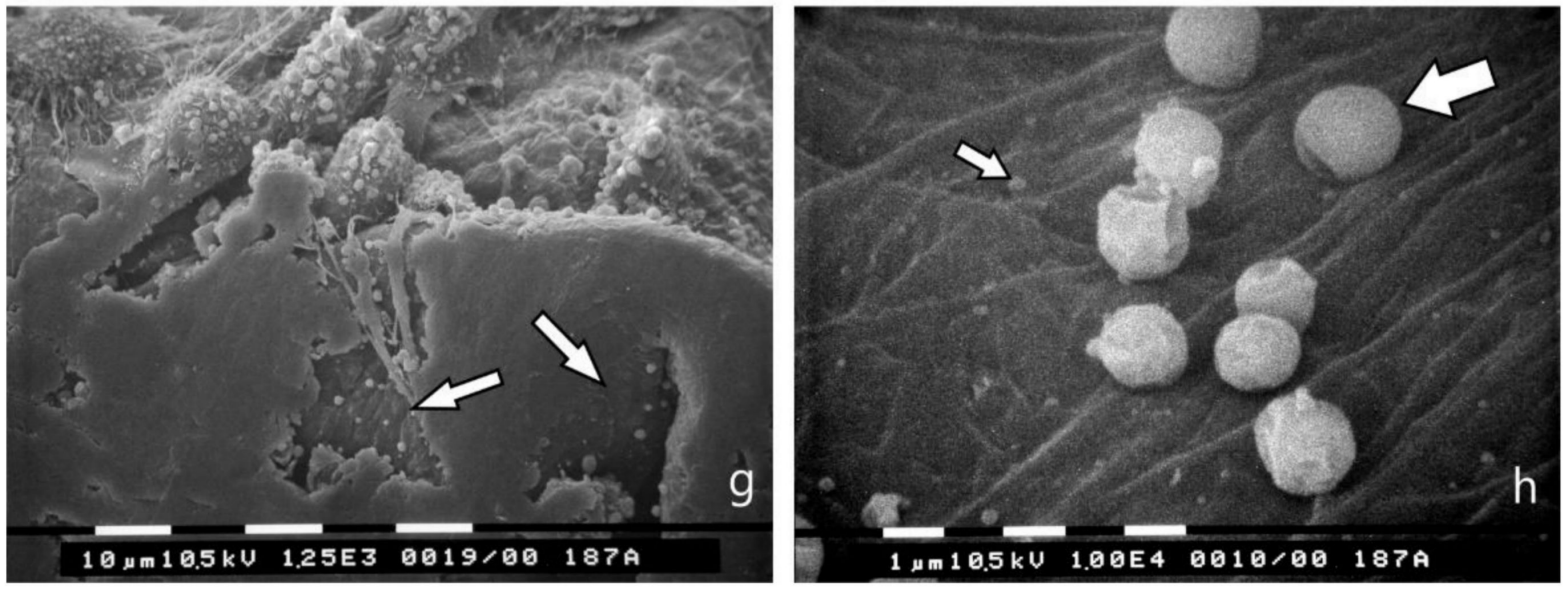
Breast cancer cells cultured on collagen type I fibers bundled parallel to the culture plane (TACS II). (**a**) Section of the collagen membrane of Achilles tendon including collagen fibers parallel to the culture plane used as substrate for culture of breast cancer cells. Bar 100 μm; (**b**) MCF-7 cells on collagen membrane appear as grouped globular/spherical cells with many microvilli but no microvesicles. Bar 100 μm; (**c**) Grouped MDA-MB-231 cells on the same collagen membrane show globular/spherical shape and elongated and spindle-like ones. Bar 100 μm; (**d**) At a higher enlargement, four MCF-7 cells show cell-cell contacts and many microvilli on their cytoplasmic surface. Bar 10 μm; (**e**) At higher enlargement the globular/spherical MDA-MB-231 cells appear covered by many microvesicles, which are very few on elongated or spindle-like cells. Bar 10 μm; (**f**) A spindle-like MDA-MB-231 cell with microvilli shows two invadopodia (arrow) degrading the collagen fibrils (easily recognizable in the lower portion of the picture). Bar 1 μm; (**g**) MDA-MB-231 cells are visible on the collagen membrane but not in the thickness of the same substrate. However, many exosomes and microvesicles (arrows) shed by MDA-MB-231 cells penetrated the superficial thickness of the collagen membrane and are visible in the inter fiber spaces. Bar 100 μm; (**h**) At higher enlargement, exosomes (small arrow) and microvesicles (large arrow) lying on collagen fibrils are clearly visible in the thickness of the collagen membrane. Bar 1 μm.

As depicted in Figure 6a, the collagen membrane of equine Achilles tendon showed fibers parallel to the smooth but undulated surface and small inter-fiber spaces in its thickness. Almost all MCF-7 cells appeared grouped and showed a globular/spherical shape with many microvilli on the cytoplasmic surface (Figure 6b,d). No cell was able to penetrate even partially through the surface of dense collagen fibers. MDA-MB-231 cells cultured on the same collagen membrane appeared grouped and showed many globular/spherical shaped cells and few elongated and spindle-like cells (Figure 6c). The globular/spherical cells were completely covered by microvesicles or blebs (Figure 6e), whereas the mesenchymal, spindle-like shaped cells showed rare microvesicles or blebs but exhibited ventral invadosomes (Figure 6f). Similar to MCF-7 cells, no MDA-MB-231 cell was able to penetrate in the thickness of the collagen membrane after 3 h and also after 24 and 36 h of culturing (unpublished data). However, in MDA-MB-231 cultures globular cytoplasmic structures resembling microvesicles and exosomes in shape and size were detectable on the collagen surface but also inside the superficial inner spaces of the membrane thickness (Figure 6g,h).

### 3.6. Bundled Type I Collagen Fibers Orthogonal to the Plane of the Culture (TACS III)

**Figure 7:**
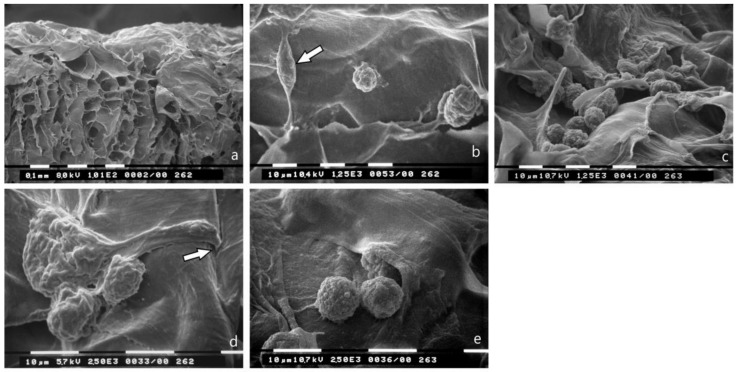

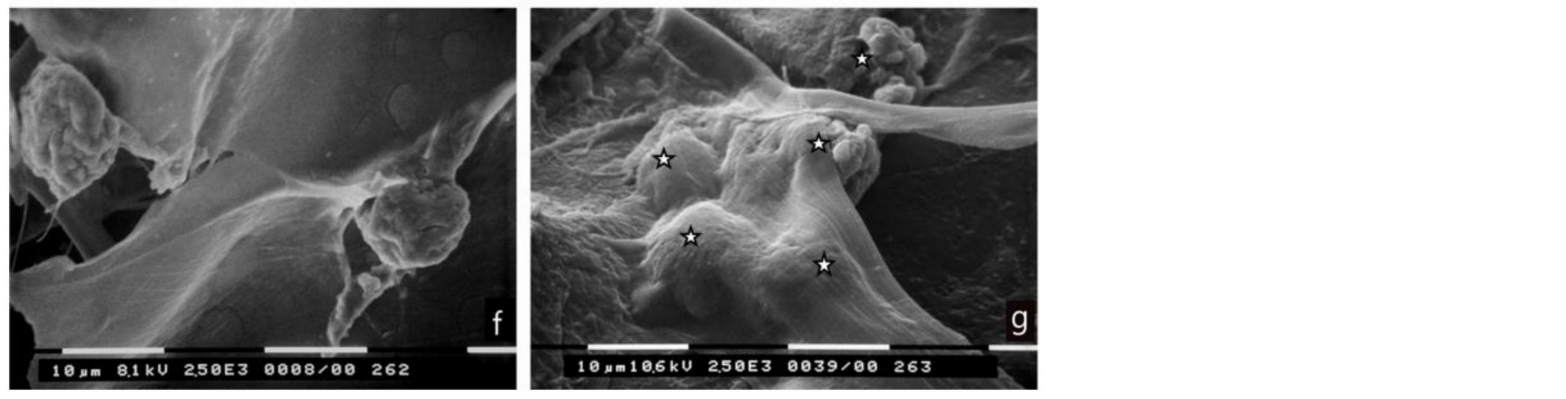
Breast cancer cells cultured on type I collagen fibers bundled orthogonal to the culture plane (TACS III). (**a**) Section of the collagen membrane with collagen fibers orthogonal to the culture plane used as substrate for cultures of breast cancer cells. Inter fiber spaces and channels are available for breast cancer invasion. (**b**) MCF-7 cells lying on the collagen membrane surface mainly show a globular/spherical shape with cytoplasmic convolutions and no microvilli (in the center and on the right), but also some spindle-like cells (arrow). Bar 10 μm. (**c**) MDA-MB-231 cells on the collagen membrane show a globular/spherical shape with microvesicles on the cell surface. Bar 10 μm. (**d**) Two globular MCF-7 cells and a flattened/elongated cell showing invadopodia penetrating the collagen membrane surface (arrow) are visible. Cytoplasmic convolutions with no microvilli are detectable on the cell surfaces. Bar 10 μm. (**e**) MDA-MB-231 cells on the collagen membrane show a globular/spherical shape with few microvesicles. Bar 10 μm. (**f**) Globular/spherical MCF-7 cells penetrated the thickness of the collagen membrane through inter fibers wide channels. Bar 10 μm. (**g**) Globular/spherical cells with few microvesicles (stars) partially or completely invaginated a superficial sheet of the collagen membrane. Bar 10 μm.

Most of the MCF-7 cells that were cultured on a collagen membrane while allowing free channels of 30–60 µm diameter among collagen fibers with different arrays but mainly orthogonally arranged to the culture plane (Figure 7a) showed both globular/spherical shapes and very few elongated, spindle-like cells (Figure 7b). No microvilli or microvesicles were visible on the MCF-7 cells surface exhibiting evident cytoplasmic convolutions. Some of them appeared invaginating by invadopodia into the surface of the membrane (Figure 7d). Globular/spherical MCF-7 cells well adhering to the membrane surface and with no microvilli or microvesicles were also detectable in the most superficial inner thickness of the membrane (Figure 7f). MDA-MB-231 cells cultured on the same collagen membrane mainly appeared as globular/spherical shaped cells rich in microvesicles or blebs (Figure 7c,e). Some of them were able to completely penetrate the superficial collagen layers (Figure 7g).

The results of SEM analysis (Figure 1, Figure 2, Figure 3, Figure 4, Figure 5, Figure 6 and Figure 7), highlighting the similarities and differences, are summarized in Table 2.

### 3.7. Substrate-Specific Invasion of MDA-MB-231 and MCF-7 Breast Cancer Cells

**Figure 8:**
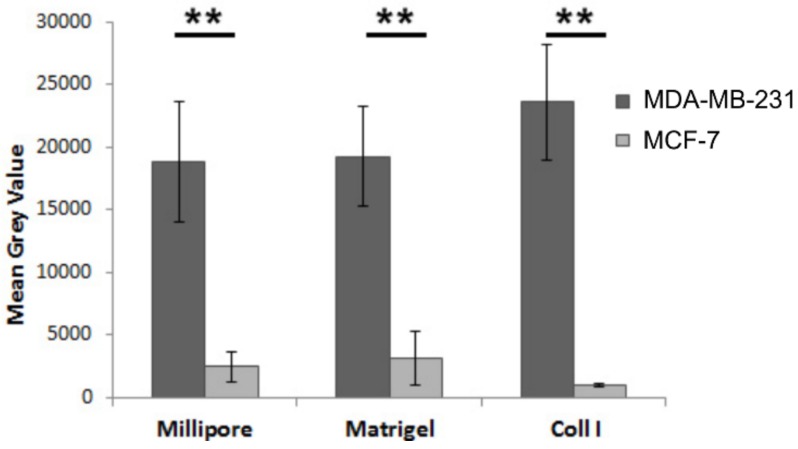
Migration of breast cancer cells cultured on different substrates. MDA-MB-231 and MCF-7 cells were seeded on Millipore filters or Millipore filters covered with Matrigel (0.18 μg/mL) or collagen type I (Coll I) and incubated for 5 h. All assays were performed as described in Materials and Methods. Asterisks (**) indicate statistically significant differences (*p* ≤ 0.01).

The ability of malignant cells to pass through the Matrigel- or type I collagen-coated filters represents a measure of their invasiveness. Therefore, an invasion assay was carried out to evaluate the epigenetic role of the different biological barriers, which cancer cells encounter during their migration in ECM, as described in the Materials and Methods section. The obtained results confirmed that the mesenchymal MDA-MB-231 cells are obviously more invasive than epithelial MCF-7 cells. However, no evident differences relating invasion ability between uncoated Millipore filter or Matrigel-coated Millipore filter groups were found both in MDA-MB-231 and MCF-7 cells. Type I collagen-coated Millipore filter increased the ability of MDA-MB-231 breast cancer cells to penetrate the membrane, as compared to uncoated Millipore filter and Matrigel-coated filter. On the other hand, collagen type I does not significantly affect MCF-7 cells’ invasive potential (Figure 8).

### 3.8. Effects of Type I Collagen on Major EMT Markers and ECM Modulators of Breast Cancer Cells

**Figure 9:**
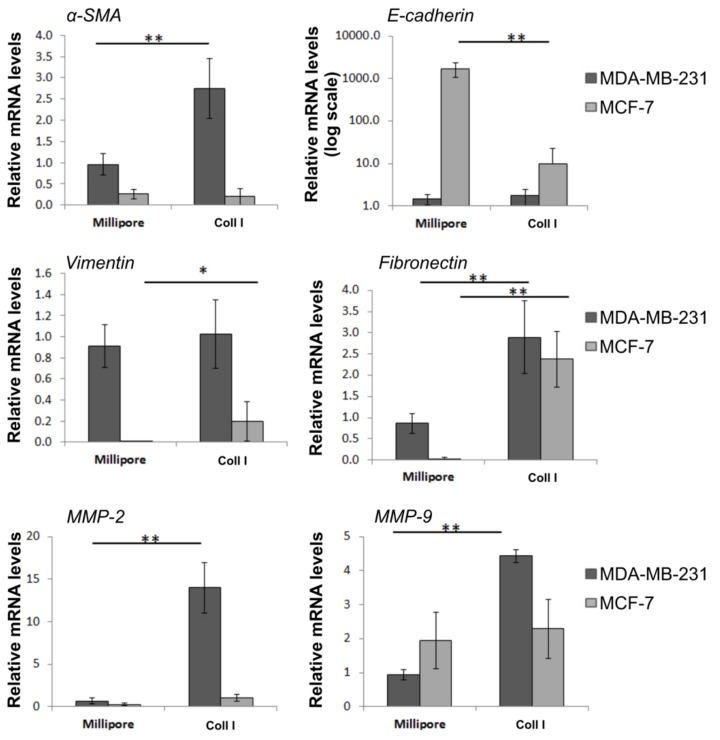
Type I collagen induces striking changes in EMT markers and significant alterations in the expression of ECM components of breast cancer cells. Real-time PCR analysis of a-SMA, E-cadherin, vimentin, fibronectin, MMP-2 and MMP-9 on MDA-MB-231 and MCF-7 cells seeded on uncoated Millipore filter or filter covered with collagen type I and incubated for 24 hours. Expression was normalized to GAPDH expression. Asterisks (*), (**) indicate statistically significant differences (*p* ≤ 0.05 and *p* ≤ 0.01, respectively).

To assess whether type I collagen may modulate EMT program in MDA-MB-231 and MCF-7 breast cancer cells, we investigated the expression levels of significant EMT markers following treatment with type I collagen (0.2 μg/μL) in a Millipore filter. Our data revealed (Figure 9) that type I collagen treatment significantly upregulated the expression of α-SMA and fibronectin, in MDA-MB 231 cells, whereas the expression of epithelial marker E-cadherin was not affected by type I collagen. On the contrary, type I collagen treatment significantly reduced E-cadherin expression in MCF-7 along with the increased levels of vimentin and fibronectin. As far as MMPs are concerned, both MMP-2 and MMP-9 expression levels are upregulated in MDA-MB 231 cells following treatment with type I collagen on Millipore filter, whereas in MCF-7 only MMP-2 seems to be slightly upregulated in type I collagen cultures.

## 4. Discussion

Cancer cell invasion and migration potential in peritumoral stroma are closely related to cell shape and cytoplasmic processes. Depending on cell type and tumor microenvironment, cells can migrate individually or collectively as multicellular groups, by ameboid or mesenchymal movement [6]. In particular, spherical-shaped cells migrate in low adhesion force or high actomyosin-mediated contractility by ameboid movement and are probably favored in travelling through wide hydrated tissue spaces or liquids, like blood [23]. On the other hand, the elongated/fusiform or spindle-like cells resulting from the EMT process usually invade by a mesenchymal movement, just by developing filopodia and invadosomes containing MMPs to directly digest the ECM.

In the present study, variable parameters in cell cultures, such as different microenvironments and orientation of the substrates, were used to highlight the impact of tumor microenvironment in cell morphology, since it is well established that the dynamic interplay among cancer cells and tumor microenvironment, including tumor stroma, mediates tumor progression through interactions between matrix macromolecules. Under physiological conditions, however, the microenvironment is different from the tumor one; thus, we focused on breast cancer cells for the examination. To this end, we utilized two breast cancer cell lines that differ in terms of aggressiveness and cell morphology: the epithelial MCF-7 cells of low aggressiveness, and the mesenchymal, tumorigenic MDA-MB-231 cells of high aggressiveness. The control cases of our study were represented by the 2D cultures on plastic flasks of the respective cells. Future studies on the evaluation of possible effects in normal epithelial cells, such as MCF-10A or HMT-S1, will further help to improve this approach.

We demonstrated that, in general, MCF-7 and MDA-MB-231 breast cancer cells cultured in 2D and 3D cultures on different collagen substrates show different phenotype at SEM: i) a globular/spherical shape, ii) a flattened polygonal shape, and iii) an elongated/fusiform and spindle-like shapes. The distribution of different cell shapes changed with the distinct collagen fiber/fibril physical array and size. Comparing the shape and surface morphology of MCF-7 and MDA-MB-231 cells in 2D cultures on polystyrene flasks relative to 3D cultures on Millipore filter, it is evident that the microenvironment of the culture ground plays a fundamental role in changing the phenotype of breast cancer cells. In fact, almost all the flattened polygonal MCF-7 cells and the flattened elongated MDA-MB-231 cells in 2D cultures lose their shape transforming into globular/spherical ones in 3D Millipore cultures.

Furthermore, we revealed that cancer cell surface morphology alters in relation to cancer cell shape. It is of interest that most of the low aggressive, round MCF-7 cells show only microvilli on the cytoplasmic surface, whereas aggressive MDA-MB-231 cells exhibit also many microvesicles which are reported to contain metalloproteases [9,45,46]. In both 2D and 3D cultures, the mesenchymal-shaped cells with a spindle-like shape usually show fewer microvilli and microvesicles and are mostly observed in MDA-MB-231 cells. Both the globular-spherical and mesenchymal shaped cells are able to pass through the 8 μm holes of the Millipore filter; the rounded ones mainly showing a collective invasion mode and the mesenchymal ones predominantly following an individual migration mode.

The first barrier that breast cancer cells have to cross in order to migrate is the epithelial basement membrane that we reproduced in vitro by the soluble basement membrane, Matrigel [44]. Comparing the pictures of MCF-7 and MDA-MB-231 cells cultured in 3D Millipore filter and in Millipore filter covered with Matrigel (0.18 μg/mL), no great differences are evident in cell shape, cell surface morphology, or invasion mode, suggesting a relatively poor resistance of the basement membrane-like sheet at a low concentration [47]. To confirm these data, the invasion test evaluating the invasive potential of MDA-MB-231 and MCF-7 cells through interaction with Matrigel did not show any difference between cells cultivated in contact with Matrigel or uncoated Millipore filter.

MCF-7 globular/spherical cells mainly show microvilli on their surface, whereas the same rounded cells in MDA-MB-231 cells exhibit also microvesicles, as described in SEM images of 3D Millipore cultures. MCF-7 cells passing through the holes of the Millipore filter show no microvesicles and very few or no microvilli, whereas the MDA-MB-231 cells on the lower surface of the Millipore filter show both microvilli and microvesicles or blebs, suggesting the relation between microvesicles, exosomes and a higher cell aggressiveness. The particular shape and size of the top of the microvilli enhances the suggestion that exosomes are directly shed by microvilli tips as suggested in previous report [45]. However, when Matrigel is added in a higher concentration (3.0 μg/mL) the ground culture appears as a compact, uniform and thicker barrier and both the MCF-7 and MDA-MB-231 cells develop invadopodia to penetrate the thicker Matrigel/basal membrane. To confirm the increased aggressiveness of breast cancer cells cultured on a thick Matrigel layer, some exosomes and microvesicles are shed by the cancer cell surface on the Matrigel ground.

At the next level, we evaluated the effects of type I collagen arrangement on the morphology of breast cancer cells. MCF-7 cells cultured on a randomly arranged collagen fibril network mimicking the sub-basement membrane definitely appear isolated, and many of them show a mesenchymal spindle-like shape and invadosomes. The limited space between collagen fibrils prevents the invasion of the globular cells moving by an ameboid movement. This condition induces a morphological epithelial-to-mesenchymal transition in MCF-7 cells, which develop invadosomes to degrade the collagen fibrils and appear very similar to the aggressive MDA-MB-231 mesenchymal cells. Although the invasive capacity of tumor cells appears to be unaffected by the presence of collagen fibril network in the invasion test, the expression levels of critical EMT markers, MMP-2 and MMP-9 have been shown to change when breast tumor cells are cultured in the presence of type I collagen. In particular, the mesenchymal markers (vimentin, fibronectin) were strongly increased in MCF-7 cells grown on a collagen fibrillar network, while the epithelial marker E-cadherin of the same cells was drastically decreased, as described by other authors [48]. Thus, these changes in the expression of genes related to EMT and ECM remodeling may be associated with an increased aggressiveness of MCF-7 cells, even though invasive ability of these cells remained unaffected.

During breast cancer progression, a rearrangement of the surrounding microenvironment, consisting of collagen deposition or desmoplasia, occurs. Collagen production is followed by a remodeling and stiffening of collagen fibers, which increases resistance to assault of proteolytic degradation by cancer cells [24,25]. Some authors have classified this first step of collagen reorganization as TACS-I [43]. In a second stage of the peritumoral stroma development, the fibrillar collagen of the dermis is arranged into collagen fibers bundled in parallel to the tumor surface, described as TACS-II [37,43,49]. We reproduced the tumor-stroma interface corresponding to the TACS-II step of ECM in vitro by culturing breast cancer cells on a type I collagen membrane of the Achilles tendon. In TACS-II, the particular array of the peritumoral collagen fibers limits the tumor mass expansion and cancer cell invasion. In fact, the orthogonal compression by tumor mass on the parallel collagen fibers evokes a shrinkage of the fibers themselves, which become much more resistant to MMP action and do not allow the development of inter-fiber spaces [31]. As described in vivo, no cell is able to cross the barrier with such a collagen array in vitro after 36 hours of culture (unpublished data). It is of interested that both MDA-MB-231 and MCF-7 globular/spherical cells respond to this compact collagen barrier by increasing microvesicles and microvilli, respectively, generating exosomes [9,45]. Although no cells pass through the collagen substrate, exosomes and microvesicles can penetrate the thickness of the upper portion of the collagen membrane with the aim of degrading ECM and creating tissue gaps favoring cell penetration only in MDA-MB-231 cultures. Notably, the mesenchymal spindle-like cells, which are observed only in MDA-MB-231 cells, develop invadopodia to attack the compact collagen barrier [23].

In a third stage of the peritumor stroma signature (TACS-III), the collagen fibrils of the dermis bundle into collagen fibers with a random array or even a radial arrangement [31,35,43]. We reproduced this stage by culturing breast cancer cells on a type I collagen membrane of Achilles tendon containing collagen fiber bundles mainly orthogonal to the culture plane, with gaps and relatively wide channels for cell migration. This sponge-like collagen array seems to influence the phenotype and behavior of both MCF-7 and MDA MB 231 cells, which are now able to cross the collagen spongy membrane. MCF-7 cells mainly demonstrate a globular/spherical shape, with evident cytoplasmic convolutions and no microvilli or microvesicles, but the presence of rare spindle-like cells lying on the collagen membrane surface indicates that a morphological EMT process occurs. Some MCF-7 globular cells invaginate into the collagen membrane surface by invadopodia, whereas other cells reach the inter-fiber channels in the thickness of the membrane. The MDA-MB-231 cells mainly appear as globular/spherical shaped cells with cytoplasmic convolutions overlapping with few microvesicles. Some of them are able to completely invaginate into the superficial layer of the collagen membrane and penetrate the inter-fiber channels. The minor presence of microvilli and microvesicles on the cancer cell surface seems to be related to the poor resistance that the cells find in the surrounding microenvironment.

In conclusion, our data show that the culture substrate can change the phenotype of breast cancer cells which show different shapes and surface morphology adapting to the chemical interactions and mechanical resistance that they meet in the surrounding 3D microenvironment. All 3D culture substrates seem to induce a 3D globular shape in breast cancer cells, which is related to the ameboid movement and it is appropriate for travelling in vasculature or lymphatic system just in relation to mechanical forces associated with fluid flow and shear [23,50]. A consistent mechanical barrier, like a thick Matrigel sheet (basement membrane), a reticular collagen fibril network, or the dense collagen fibers with different arrays are related to the development of invadopodia, which represents the first cell adjustment to start a proteolytic mesenchymal invasion in both MCF-7 and MDA-MB-231 cells. When breast cancer cells find narrow spaces to penetrate the stiff network of type I collagen single fibrils randomly arranged (lamina propria), even the low aggressive MCF-7 cells show an EMT with flattened-elongated cells, invadopodia and long cytoplasmic processes, which are able to adhere to collagen by integrins and to penetrate by a mesenchymal movement into modified fibrillar gaps [51,52,53]. Both MCF-7 and MDA-MB-231 elongated cells showed few microvilli and microvesicles, which are instead abundant in globular/spherical shaped cells. This is mainly evident when the dense collagen fibers run parallel to the tumor surface, thus representing a valid barrier to the cancer cell invasion. In this situation, we observed that the MCF-7 globular cells responded by producing more microvilli, and MDA-MB-231 cells exhibited a lot of microvesicles, which were also detectable together with exosomes in the inter-fiber spaces. Globular/spherical cells tend to move by ameboid invasion in the absence of proteolytic ECM breakdown, but in absence of natural gates in the ECM, they probably react by producing many more exosomes and microvesicles, which are an expression of high cell aggressiveness [54,55,56]. It is well established that the extracellular vesicles containing membrane-type 1 MMP (MT1-MMP), sialidase and heparanase on their surface are able to invade the dense stroma by degrading ECM microenvironment [46,57]. In confirmation of this, when the collagen array offers wide channels or tissue gaps wide enough to favor an easy ameboid invasion of breast cancer cells, neither the most abundant globular nor the few spindle-like MCF-7 cells lying on the collagen membrane surface show any microvilli. Similarly, the more aggressive MDA-MB-231 cells exhibit very few microvesicles on cytoplasmic surface. Comparing the parallel to orthogonal collagen fiber array proposed in vitro, which correspond to TACS-II and TACS-III in vivo, we can conclude that ECM plays dual roles as a tumor suppressor at the early stages, but paradoxically also as a tumor promoter in the later stages of tumor progression [31].

In particular, these data suggest that the different collagens and fibrillar collagen array in 3D cultures of breast cancer cells modify cell shape, sometimes inducing EMT and modulating the extracellular vesicles production. This in vitro model will be used to further investigate the effects on normal breast epithelium and the biochemical roles of ECM macromolecules in limiting or favoring cancer invasion of tumor stroma in vivo.

## 5. Conclusions

Breast cancer cells (MCF-7 and MDA-MB-231) cultivated in 3D cultures change their phenotype as compared to 2D cultures. When breast cancer cells seeded in 3D cultures come into direct contact with different collagens, they change their phenotype and extravesicle shedding activity. Specifically, MCF-7 grown on uncoated Millipore filter and Matrigel-coated Millipore filter demonstrate a globular/spherical shape and superficial microvilli (related to exosomes shedding), whereas elongated, fusiform and globular/spherical MDA-MB-231 cells contain few microvilli but many microvesicles. Increasing the thickness of Matrigel substrate, both MCF-7 and MDA-MB-231 cells exhibit more invadosomes, which facilitate increased cell invasion. EMT is induced when MCF-7 cells are in contact with type I collagen network. Dense collagen fibers, parallel to the culture plane, do not allow the invasion of MCF-7 and MDA-MB-231 breast cancer cells, which demonstrate increased number of microvilli and microvesicles; however, only exosomes and microvesicles from MDA-MB-231 cells can penetrate the thickness of the collagen membrane. On the other hand, collagen fibers orthogonally arranged to the culture plane, allow cell invasion both in MCF-7 and in MDA-MB-231 cells. These data demonstrate the critical importance of matrix network composition and structure on breast cancer cell morphology and invasion as well as on gene expression of matrix mediators implicated in cancer progression.

## Figures and Tables

**Table 1:** List of real-time qPCR primers used in this study.

Gene	Primer Sequence
α-SMA	R: CATCAGGCAACTCGTAACTC
	F: TACTACTGCTGAGCGTGAGA
E-cadherin	R: TGGCTCAAGTCAAAGTCCTG
	F: TTCTGCTGCTCTTGCTGTTT
Fibronectin	R: GACGCTTGTGGAATGTGTCG
	F: GTGTGTTGGGAATGGTCGTG
Vimentin	R: CACTTTGCGTTCAAGGTCAAGAC
	F: AAAACACCCTGCAATCTTTCAGA
MMP-2	R: CACGCTCTTCAGACTTTGGTTCT
	F: GCGGCGGTCACAGCTACTT
MMP-9	R: CCACCCGAGTGTAACCATAGC
	F: CCTGGAGACCTGAGAACCAATC
GAPDH	R: GGAGTCCACTGGCGTCTT
	F: AGGCTGTTGTCATACTTCTCAT

**Table 2:** The most important similarities and differences of MCF-7 and MDA-MB-231 breast cancer cells cultured in different conditions are summarized.

	MCF-7 Breast Cancer Cells	MDA-MB-231 Breast Cancer Cells
Cell cultures on polystyrene flasks	Mostly grouped cells with many cell-cell contacts Flattened, smoothly polygonal cells Very few microvilli and rare cytoplasmic vesicles	Isolated cells with very few cell-cell contacts Globular/spherical cells - Flattened elongated cells - Spindle-like cells Cytoplasmic vesicles
Cell cultures on Millipore filter	Grouped globular/spherical cells Few microvilli and no microvesicles	Mostly isolated globular/spherical cells with microvesicles and few elongated or spindle-like cells with few microvilli and few microvesicles
Cell cultures on Millipore coated with Matrigel (0.18 μg/mL)	Grouped globular/spherical cells Microvilli	Mostly globular/spherical cells with microvesicles and few elongated or spindle-like cells with few microvilli and few microvesicles
Cell cultures on Millipore covered with Matrigel (3.0 μg/mL)	Grouped globular/spherical cells Few microvilli and few microvesicles Invadopodia	Many globular/spherical cells and few elongated cells Few microvilli and few microvesicles Invadopodia
Cell cultures on Millipore covered with collagen type I collagen fibrils	Cell proliferation - Isolated and relatively grouped cells Globular/spherical cells with microvesicles and flattened elongated cells Invadopodia	Cell proliferation - Isolated and relatively grouped cells Globular/spherical cells with microvesicles and flattened elongated cells Invadopodia and invadosomes
Cell cultures on type I collagen fibers bundled parallel to the culture plane (TACS II)	Grouped cells Mostly globular/spherical cells with many microvilli No cells or exosomes and microvesicles into the thickness of the collagen membrane	Relatively grouped cells Many globular/spherical cells with many microvilli and microvesicles. Few elongated and spindle-like cells with invadopodia and invadosomes No cells into the thickness of the collagen membrane but exosomes and microvesicles inside the superficial inner spaces of the collagen membrane
Cell cultures on type I collagen fibers bundled orthogonal to the culture plane (TACS III)	Mostly globular/spherical cells with cytoplasmic circonvolutions and very few elongated and spindle-like cells with invadopodia Cells invaginate the superficial sheet of the collagen membrane	Mainly globular/spherical cells with many microvesicles and few elongated cells Cells invaginate the superficial sheet of the collagen membrane
